# Qualitative clock-drawing errors across dementia etiologies and mild cognitive impairment: a clinically interpretable six-class framework

**DOI:** 10.1017/S1092852926101072

**Published:** 2026-06-18

**Authors:** Ankur Banik, Sandip Pal, Asutosh Pal, Moukoli Pal, Indranil Dutta, Dilip Roy, Amrit Chattopadhyay, Julian Benito-Leon

**Affiliations:** 1 Department of Neurology, Medical College Kolkata, Kolkata, India; 2 Department of Neurology, 12 de Octubre University Hospital, Madrid, Spain; 3 Group of Neurodegenerative Diseases, Hospital Universitario 12 de Octubre Research Institute (imas12), Madrid, Spain; 4 Network Center for Biomedical Research in Neurodegenerative Diseases (CIBERNED), Madrid, Spain; 5 Department of Medicine, Faculty of Medicine, Complutense University of Madrid, Madrid, Spain

**Keywords:** Clock drawing test, cognitive screening tools, dementia etiologies, mild cognitive impairment, qualitative error analysis

## Abstract

**Objective:**

To characterize qualitative Clock Drawing Test (CDT) error profiles across dementia etiologies and mild cognitive impairment (MCI), and to propose a clinically interpretable six-class framework.

**Methods:**

In a hospital-based study in Kolkata, India, consecutive adults with cognitive impairment completed a free-drawn “ten past ten” CDT. Errors were coded using classical qualitative categories and clock components (face, numbers, hands), then collapsed into six classes: conceptual, stimulus-bound/perseveration, spatial, planning, number-related, and graphic-conceptual. For nonexclusive domains, omnibus Pearson χ² tests summarized error distributions across diagnoses; Cramér’s V quantified effect size.

**Results:**

Participants included Alzheimer’s disease (AD; *n* = 36), vascular dementia (VaD; *n* = 16), behavioral variant frontotemporal dementia (bvFTD; *n* = 9), MCI (*n* = 19), and other conditions (*n* = 22). Although 50.0% drew a normal clock face, only 11.8% achieved perfect numbering and 12.7% set the hands correctly. Conceptual errors were most frequent (70.6%), followed by spatial errors (47.1%); neglect and counterclockwise numbering were rare (3.9% each). Error distributions differed by diagnosis for face, numbers, and hands (all *p* < 0.001; *V* = 0.4–0.5). The six-class scheme retained a significant distributional association with diagnosis (χ² = 43.365, *p* = 0.002; *V* = 0.3): bvFTD showed prominent conceptual and graphic-conceptual failures, AD combined conceptual and spatial errors, MCI emphasized spatial and number-related errors, and VaD was heterogeneous.

**Conclusions:**

Qualitative CDT profiles vary meaningfully across cognitive disorders. This concise six-class framework captures clinically salient patterns, especially in severely degraded drawings, and may complement brief memory screening and digital CDT metrics.

## Introduction

The clock drawing test (CDT) is a brief, equipment-free cognitive screening tool that probes several interacting domains, including executive control, visuospatial organization, semantic knowledge, working memory, numerical concepts, and motor planning.^1^ ^–^^3^ Although CDT performance is useful in dementia screening, its interpretation remains challenging because numerous scoring systems exist and qualitative errors often co-occur within the same drawing.^1^ ^–^^5^

These challenges are particularly relevant in mild cognitive impairment (MCI) and in clinically heterogeneous dementia cohorts, where global pass/fail scoring may obscure diagnostically informative patterns. CDT performance is also influenced by age, education, and cultural familiarity with analog clocks, making simple cutoffs difficult to apply across settings.^1^ ^–^^3^ Evidence from eastern India remains limited, further underscoring the need for pragmatic, clinically interpretable, and empirically grounded qualitative frameworks.

Accordingly, we examined qualitative CDT error profiles across patients with major neurocognitive disorders and MCI in a real-world Indian cohort. Guided by established qualitative CDT frameworks^4^ ^,^^5^ and by the observed clustering of errors, we also propose a simplified six-class scheme intended to improve bedside interpretability, particularly when drawings are severely degraded or contain overlapping error types.

## Methods

### Design and setting

We conducted a hospital-based cross-sectional observational study in the Department of Neurology (Cognitive Clinic and inpatient services) at Medical College and Hospital, Kolkata (West Bengal, India), from September 2023 through August 2024. The Institutional Ethics Committee approved the protocol, and written informed consent was obtained from each participant or a legally authorized representative.

### Participants

Consecutive adults presenting with cognitive complaints were eligible if they could cooperate with testing and provided informed consent. Exclusion criteria comprised premorbid major psychiatric disorders; serious visual or auditory impairment or language-comprehension disturbance; lack of knowledge of analog clock reading; and gross motor deficits that would preclude drawing. Demographic and clinical variables were recorded as part of the routine cognitive evaluation, including years of formal education, educational attainment category, Mini-Mental State Examination (MMSE),^6^ Clinical Dementia Rating (CDR),^7^ and instrumental activities of daily living. The Montreal Cognitive Assessment (MoCA) and Addenbrooke’s Cognitive Examination III (ACE-III) were not administered uniformly across the cohort and, therefore, were not included in the present analysis.

### Clock drawing test administration

Participants were instructed: “Please draw a clock, place all the numbers, and set the time to ten past ten.” Drawings were produced freehand on blank paper without a predrawn circle and without corrective cueing during performance. The instruction was intentionally brief, uniform, and easy to administer, following the same standardization principle used in structured CDT protocols such as that described by Cacho et al.^8^ We selected a fixed “ten past ten” target because it requires representation of two distinguishable hands, avoids hand overlap, and preserves an open clock face for qualitative inspection. Although this time setting is less conventional than some previously used CDT commands, it was applied consistently across all participants to support reproducible qualitative coding.

### Qualitative error coding

Errors were coded using eight widely cited qualitative categories (per Cahn and Freedman)^4^ ^,^^5^: stimulus-bound response (eg, minute hand toward “10” rather than “2”); conceptual deficit (semantic or rule-based failures about clock features or time setting); perseveration (repetitions or > 12 numbers/elements); left hemispace neglect (numbers crowded to the right hemispace); planning deficit (misplacement of anchor numerals 12–3–6–9); nonspecific spatial error (mis-spacing without a specific pattern); numbers outside the clock; and counterclockwise numbering. In parallel, we recorded component-specific errors for the clock face (normal; mild distortion; gross distortion), the numbers (normal; no numbers; missing; added; spatial errors; numbers outside; counterclockwise; other/multiple languages), and the hands (normal; no hands; single hand; two hands with improper representation; additional hands).

### Diagnostic ascertainment

Etiologic adjudication was supported by routine clinical investigation, including noncontrast 1.5-T brain magnetic resonance imaging (MRI) performed according to the institutional dementia protocol, which comprised three-dimensional T1-weighted volumetric sequences, fluid-attenuated inversion recovery, and susceptibility-weighted imaging. Positron emission tomography (PET) combined with computed tomography imaging was performed in selected diagnostically challenging cases.

Cognitive impairment and etiology were assigned according to the Diagnostic and Statistical Manual of Mental Disorders, Fifth Edition (DSM-5) (Mild and Major Neurocognitive Disorder),^9^ with etiologic adjudication according to the consensus criteria: National Institute on Aging–Alzheimer’s Association (NIA-AA) for Alzheimer’s disease (AD),^10^ National Institute of Neurological Disorders and Stroke–Association Internationale pour la Recherche et l’Enseignement en Neurosciences (NINDS-AIREN) for vascular dementia (VaD), and international criteria for behavioral variant frontotemporal dementia (bvFTD).^11^ Mild cognitive impairment (MCI) was defined according to the NIA–Alzheimer’s Association core clinical criteria.^12^ For clarity of reporting, diagnoses were grouped as AD, VaD, bvFTD, MCI, or other etiologies.

### Statistics

Analyses were conducted using IBM SPSS package for Windows, version 23.0. Categorical outcomes are presented as counts and percentages. Continuous and ordinal variables used to describe the cohort, including years of education, MMSE, and CDR, are summarized as medians and interquartile ranges. Between-diagnosis differences in these variables were examined descriptively using the Kruskal–Wallis test, with Dunn post hoc comparisons where appropriate. Between-group differences in categorical variables were examined using Pearson’s *χ* ^2^ tests (two-sided *α* = 0.05). Performance was evaluated at three levels: component-wise (face, numbers, hands), the eight classical qualitative categories, and a simplified six-class scheme derived from observed error clustering.

For error domains that are not mutually exclusive (numbers, hands, and classical/six-class frameworks), we constructed multiple-response contingency tables and applied *χ* ^2^ tests to compare the distribution of error occurrences across diagnoses; these omnibus tests are interpreted descriptively as pattern summaries rather than tests on exclusive patient categories. We report Cramér’s *V* as an effect size for each omnibus comparison.

## Results

### Sample and etiologies

In the 102 participants, AD constituted 35.3% (*n* = 36), VaD 15.7% (*n* = 16), bvFTD 8.8% (*n* = 9), MCI 18.6% (*n* = 19), and “other” etiologies 21.6% (*n* = 22; atypical AD, dementia with Lewy bodies, Creutzfeldt–Jakob disease, progressive supranuclear palsy, and Huntington’s disease). These proportions are consistent with reported patterns in urban Indian community and clinic samples, where AD predominates, although the burden of VaD remains substantial.^13^

Educational attainment ranged from illiteracy to graduate-level education and beyond. Median years of formal education were 10.0 in AD, 8.5 in VaD, 4.0 in bvFTD, 12.0 in MCI, and 7.0 in the Other etiologies group. High-school and graduate-level education were the most common categories overall (41/102 [40.2%] and 37/102 [36.3%], respectively), and educational distribution differed modestly across diagnostic groups (*χ* ^2^=21.621, df = 12, *p* = 0.042). Global cognitive severity measures also distinguished the MCI group from the major neurocognitive disorder groups: Median MMSE scores were 19.5 in AD, 22.0 in VaD, 13.0 in bvFTD, 27.0 in MCI, and 17.0 in the Other etiologies group, whereas median CDR scores were 1.0, 1.0, 2.0, 0.5, and 2.0, respectively. Kruskal–Wallis tests showed between-diagnosis differences for both MMSE and CDR (both *p* < 0.001), driven primarily by the MCI group. These descriptive data indicate that the MCI group was globally less impaired, while severity overlap remained among the dementia groups.

### Clock face

**Figure 1.**
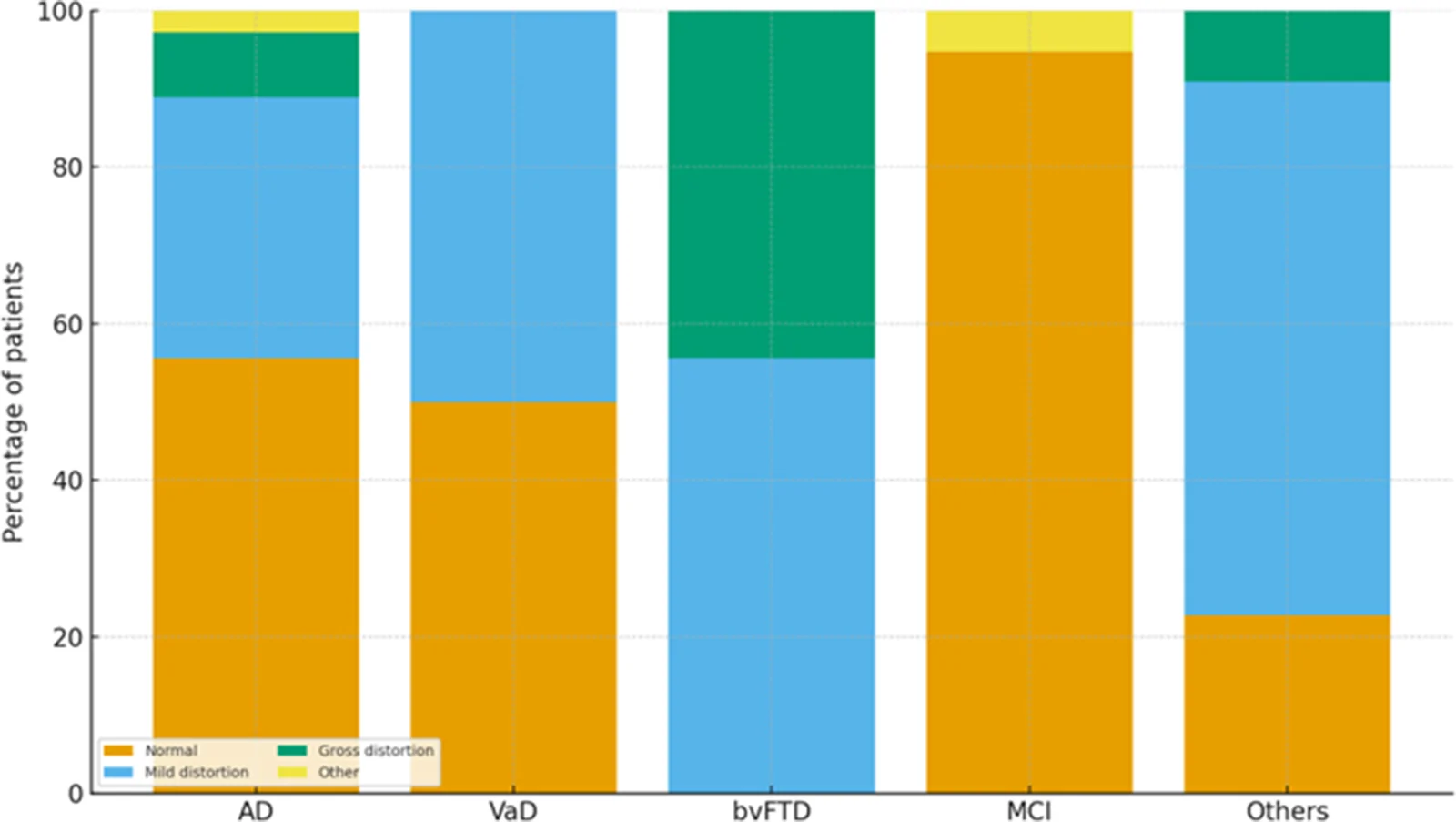
Clock face outcomes by diagnosis. Notes: 100% stacked bar chart showing the proportion of patients within each diagnosis who produced a Normal, Mild distortion, Gross distortion, or Other (noncircular/incomplete) clock face (order from left to right: AD, VaD, bvFTD, MCI, Others; total *n* = 102). Percentages are calculated within the diagnosis. Between-group differences were significant (*χ* ^2^= 47.306, df = 12, *p* < 0.001). “Other” includes a rectangular clock (AD) and an incomplete outline (MCI). Abbreviations: AD, Alzheimer’s disease; bvFTD, behavioral variant frontotemporal dementia; MCI, mild cognitive impairment; VaD, vascular dementia. Figure 1. long description.

Half of the cohort drew a normal clock face (51/102; 50.0%), 39.2% (40/102) showed mild distortion, and 8.8% (9/102) showed gross distortion; two drawings (2.0%) were noncircular/incomplete (one rectangular clock in AD; one incomplete outline in MCI). According to diagnosis, MCI preserved a normal face in 18/19 (94.7%), AD showed a mixed profile (normal 55.6%, mild 33.3%, gross 8.3%, other 2.8%), VaD split evenly between normal and mild (50.0%/50.0%), and bvFTD produced no normal faces (mild 55.6%, gross 44.4%). Between-group differences were significant (*χ* ^2^=47.306, df = 12, *p* < 0.001; Cramér’s *V* = 0.4) (Table 1 and Figure 1).

**Table 1.** Clock Face Performance by Diagnosis (Row-Percentages) Table 1. long description.

Diagnosis (*n*)	Normal	Mild distortion	Gross distortion	Other
AD (36)	20 (55.6%)	12 (33.3%)	3 (8.3%)	1 (2.8%)
VaD (16)	8 (50.0%)	8 (50.0%)	0 (0.0%)	0 (0.0%)
bvFTD (9)	0 (0.0%)	5 (55.6%)	4 (44.4%)	0 (0.0%)
MCI (19)	18 (94.7%)	0 (0.0%)	0 (0.0%)	1 (5.3%)
Others (22)	5 (22.7%)	15 (68.2%)	2 (9.1%)	0 (0.0%)

Values are *n* (row %). Categories are mutually exclusive (percentages sum to ~100% within each diagnosis). Pearson’s *χ* ^2^ compares distributions across diagnoses (*χ* ^2^ =47.306, df = 12, *p* < 0.001; Cramér’s *V* = 0.4). “Other” denotes a non-circular or incomplete outline (one rectangular clock in AD; one incomplete outline in MCI). Abbreviations: AD, Alzheimer’s disease; bvFTD, behavioral variant frontotemporal dementia; MCI, mild cognitive impairment; VaD, vascular dementia.

### Numbers

**Figure 2.**
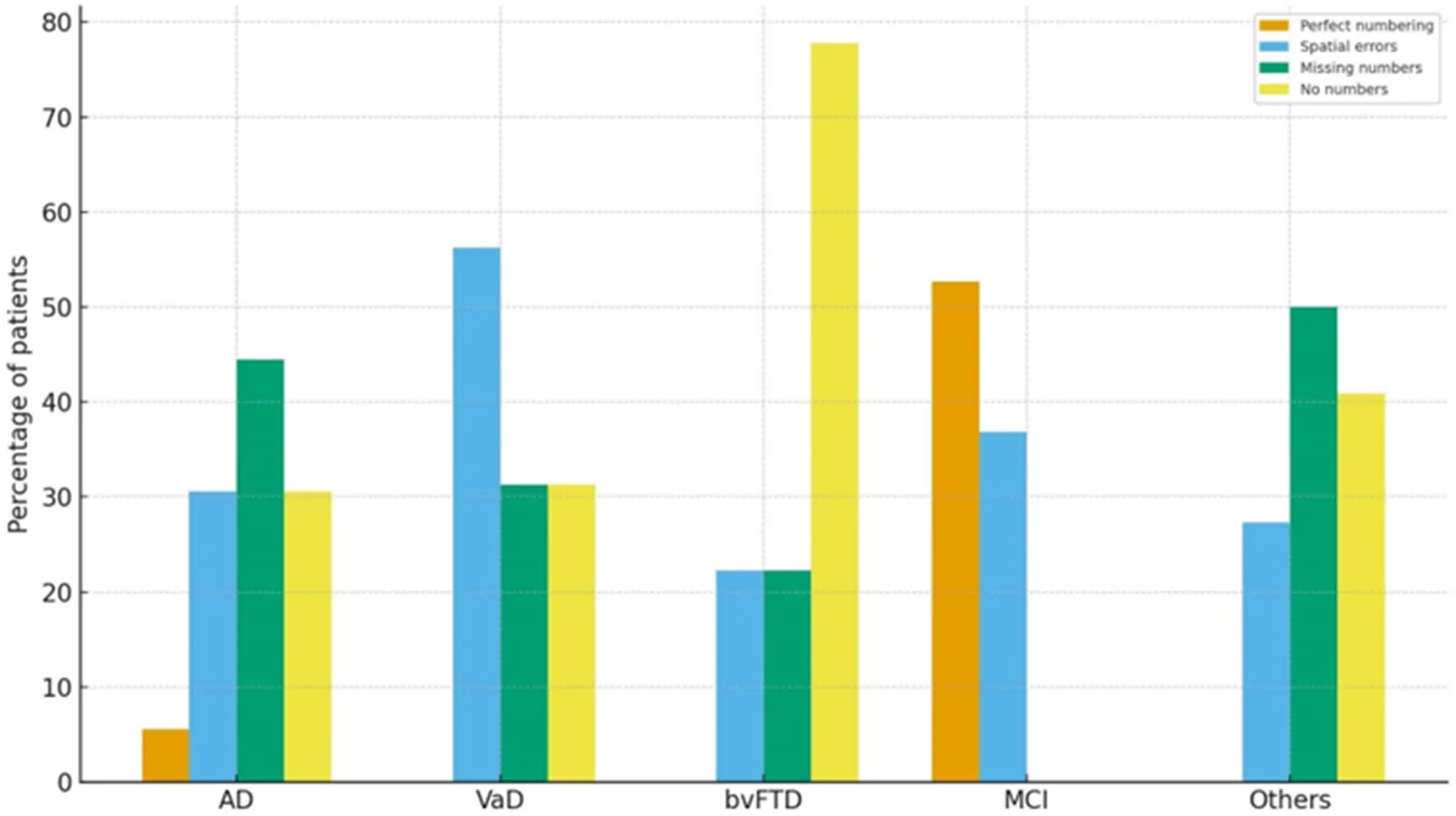
Key number-related outcomes by diagnosis (non–mutually exclusive). Notes: Grouped bar chart showing Perfect numbering (all numerals 1–12, ordered/appropriately spaced), Spatial errors (spacing/ordering errors not due to anchor misplacement or neglect), Missing numbers, and No numbers for each diagnosis (order left-to-right: AD, VaD, bvFTD, MCI, Others; total *n* = 102). Percentages are within diagnosis; categories are non–mutually exclusive, so sums across bars can exceed 100%. Between-group differences were significant (*χ* ^2^ = 82.951, df = 28, *p* < 0.001). Figure 2. long description.

Only 12/102 (11.8%) achieved perfect numbering. The most frequent number-related errors were spatial (35/102; 34.3%), missing numbers (34/102; 33.3%), and no numbers (32/102; 31.4%). Perfect numbering occurred predominantly in MCI (10/12), while a conspicuous bvFTD pattern had no numbers (7/9). The distribution of number-related error occurrences differed by diagnosis (*χ* ^2^=82.951, df = 28, *p* < 0.001; Cramér’s *V* = 0.5) (Table 2 and Figure 2). Because the number of error categories is nonexclusive, the *χ* ^2^ statistic is interpreted as a distributional summary across diagnoses (see Statistics).

**Table 2.** Number-Related Performance by Diagnosis (Row-Percentages; Nonexclusive) Table 2. long description.

Diagnosis (*n*)	No numbers	Missing numbers	Added (>12)	Spatial errors	Counterclockwise	Outside the circle	Other/multiple language	Perfect numbering
AD (36)	11 (30.6%)	16 (44.4%)	3 (8.3%)	11 (30.6%)	2 (5.6%)	10 (27.8%)	4 (11.1%)	2 (5.6%)
VaD (16)	5 (31.3%)	5 (31.3%)	1 (6.3%)	9 (56.3%)	1 (6.3%)	4 (25.0%)	2 (12.5%)	0 (0.0%)
bvFTD (9)	7 (77.8%)	2 (22.2%)	0 (0.0%)	2 (22.2%)	0 (0.0%)	0 (0.0%)	0 (0.0%)	0 (0.0%)
MCI (19)	0 (0.0%)	0 (0.0%)	0 (0.0%)	7 (36.8%)	1 (5.3%)	3 (15.8%)	0 (0.0%)	10 (52.6%)
Others (22)	9 (40.9%)	11 (50.0%)	2 (9.1%)	6 (27.3%)	0 (0.0%)	2 (9.1%)	1 (4.5%)	0 (0.0%)
Total (102)	32	34	6	35	4	19	7	12

Values are *n* (row %). Categories are not mutually exclusive (except “Perfect numbering”); within-diagnosis percentages can exceed 100%. “Perfect numbering” = numerals 1–12 present, in order, and reasonably spaced. “Spatial errors” = spacing/ordering errors not attributable to anchor misplacement or neglect. Counterclockwise numbering is rare (4/102; 3.9%). Pearson’s *χ* ^2^ summarizes distributional differences in error occurrence across diagnoses (*χ* ^2^ = 82.951, df = 28, *p* < 0.001; Cramér’s *V* = 0.5). The Total row reports raw counts (no percentages). Abbreviations: AD, Alzheimer’s disease; bvFTD, behavioral variant frontotemporal dementia; MCI, mild cognitive impairment; VaD, vascular dementia.

At the diagnosis level, more than half of patients with MCI showed perfect numbering (10/19; 52.6%), followed by a majority of spatial errrors (7/19) and a few outside-the-clock errors (3/19). In bvFTD, absence of numbers was striking (7/9; 77.8%), with a few other errors. AD showed a mixed profile—missing (16/36) and spatial (11/36) errors were frequent, with no numbers also common (11/36). VaD leaned toward spatial errors (9/16; 56.3%), with missing and no numbers in each of the 5/16. The heterogeneous Other etiologies group was dominated by missing (11/22), spatial (6/22), and no-number (9/22) entries. Counterclockwise numbering was rare overall (4/102; 3.9%).

### Hands

**Figure 3.**
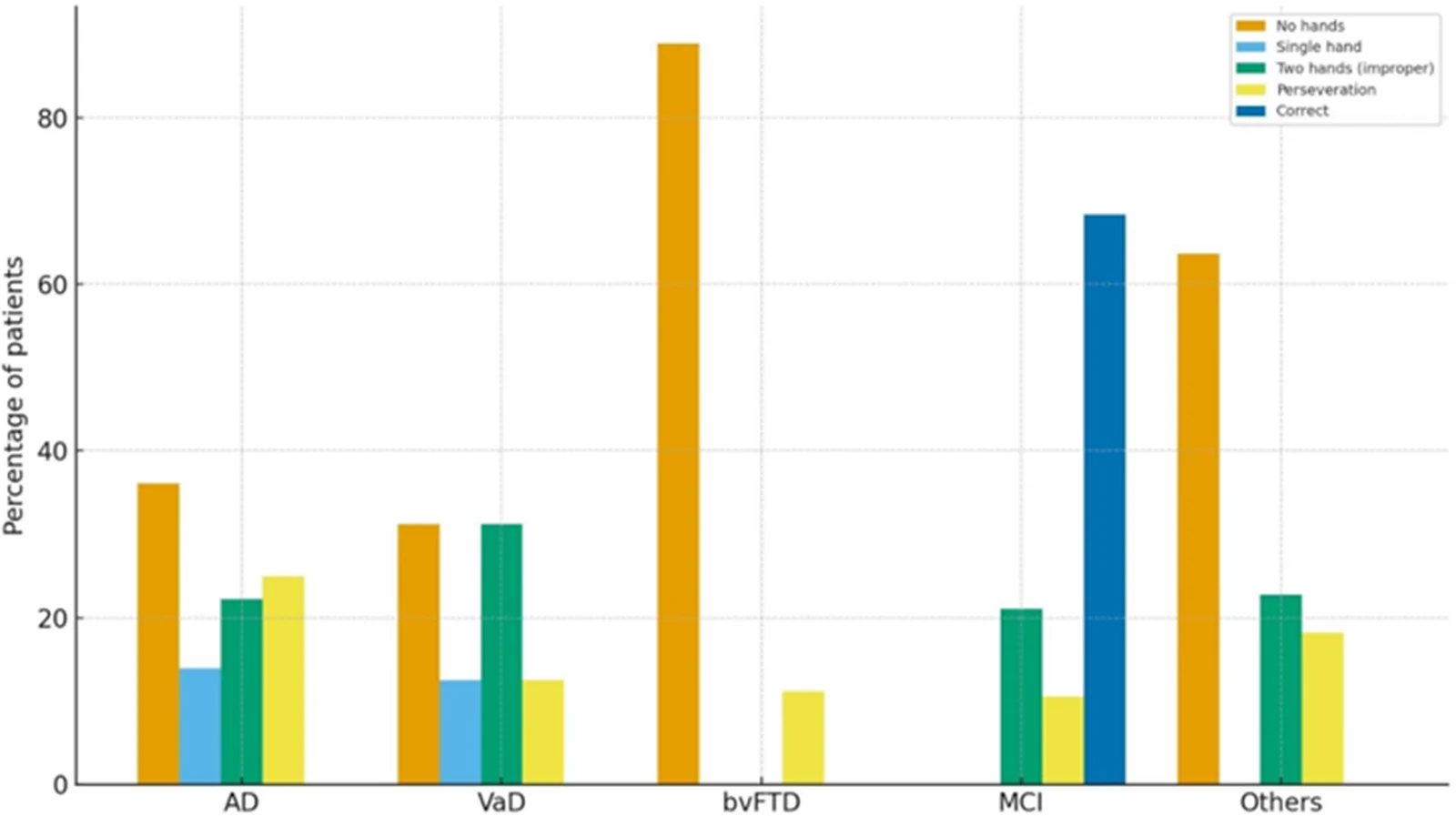
Hand-setting outcomes by diagnosis. Notes: Grouped bar chart showing No hands, Single hand, Two hands (improper), Perseveration (extra hands), and Correct hand placement for each diagnosis (order left-to-right: AD, VaD, bvFTD, MCI, Others; total *n* = 102). Percentages are within the diagnosis. Between-group differences were significant (*χ* ^2^ = 83.618, df = 16, *p* < 0.001). Correct hands were observed only in MCI. Minor row inconsistencies in the source table reflect how hand features were recorded for some drawings; interpret category rates individually. Figure 3. long description.

Only 13/102 (12.7%) set the hands correctly. The most common finding was no hands (40/102; 39.2%), followed by two hands incorrect (22/102; 21.6%), perseveration (18/102; 17.6%), and single hand (7/102; 6.9%). Correct hands appeared exclusively in MCI (13/19; 68.4%); 8/9 bvFTD omitted hands entirely. The distribution of hand-related error occurrences differed by diagnosis (*χ* ^2^ = 83.618, df = 16, *p* < 0.001; Cramér’s *V* = 0.5) (Table 3 and Figure 3). Hand categories are nonexclusive; minor row inconsistencies reflect overlapping codes and a small number of incomplete annotations—which require interpretation of individual category rates (see Table 3 footnote).

**Table 3.** Hand-Setting Performance by Diagnosis (Row-Percentages; Nonexclusive) Table 3. long description.

Diagnosis (*n*)	No hands	Single hand	Two hands (improper)	Perseveration	Correct
AD (36)	13 (36.1%)	5 (13.9%)	8 (22.2%)	9 (25.0%)	0 (0.0%)
VaD (16)	5 (31.3%)	2 (12.5%)	5 (31.3%)	2 (12.5%)	0 (0.0%)
bvFTD (9)	8 (88.9%)	0 (0.0%)	0 (0.0%)	1 (11.1%)	0 (0.0%)
MCI (19)	0 (0.0%)	0 (0.0%)	4 (21.1%)	2 (10.5%)	13 (68.4%)
Others (22)	14 (63.6%)	0 (0.0%)	5 (22.7%)	4 (18.2%)	0 (0.0%)

Values are *n* (row %). Categories are not mutually exclusive; within-diagnosis percentages may not sum to 100%. A small number of drawings had incomplete hand annotations, so row totals can be slightly < *n* in some diagnoses—interpret category rates individually. “Perseveration” denotes ≥3 hands or repeated/extra hands; “Two hands (improper)” denotes two hands drawn but incorrect time and/or no length differentiation. Correct hand placement occurred only in the MCI group. Pearson’s *χ* ^2^ summarizes distributional differences in error occurrence across diagnoses (*χ* ^2^ =83.618, df = 16, *p* < 0.001; Cramér’s *V* = 0.5). Abbreviations: AD, Alzheimer’s disease; bvFTD, behavioral variant frontotemporal dementia; MCI, mild cognitive impairment; VaD, vascular dementia.

### Eight classical qualitative error categories

Using the classical categories (Freedman/Cahn), conceptual errors were most frequent (72/102; 70.6%), followed by spatial (48/102; 47.1%). Neglect and counterclockwise numbering were each uncommon (4/102; 3.9%). Conceptual errors predominated in AD, VaD, and bvFTD, whereas spatial errors were most common in MCI. Because categories are nonexclusive, the *χ* ^2^ comparison is interpreted as a distributional difference in error occurrences across diagnoses (Table 4).

**Table 4.** Classical Qualitative Error Categories (Cahn/Freedman) by Diagnosis (Row-Percentages; Nonexclusive) Table 4. long description.

Diagnosis (*n*)	Conceptual	Perseveration	Stimulus-bound	Planning	Spatial	Hemineglect	Numbers outside	Counterclockwise
AD (36)	32 (88.9%)	10 (27.8%)	4 (11.1%)	10 (27.8%)	20 (55.6%)	0 (0.0%)	10 (27.8%)	2 (5.6%)
VaD (16)	10 (62.5%)	3 (18.8%)	1 (6.3%)	6 (37.5%)	9 (56.3%)	2 (12.5%)	4 (25.0%)	1 (6.3%)
bvFTD (9)	9 (100.0%)	2 (22.2%)	0 (0.0%)	0 (0.0%)	2 (22.2%)	1 (11.1%)	0 (0.0%)	0 (0.0%)
MCI (19)	0 (0.0%)	2 (10.5%)	1 (5.3%)	0 (0.0%)	7 (36.8%)	0 (0.0%)	3 (15.8%)	1 (5.3%)
Others (22)	21 (95.5%)	8 (36.4%)	0 (0.0%)	10 (45.5%)	10 (45.5%)	1 (4.5%)	2 (9.1%)	0 (0.0%)

Values are *n* (row %). Categories are not mutually exclusive; within-diagnosis percentages can exceed 100% (each column reflects the presence of that error in a drawing, and a patient may exhibit multiple errors). Definitions: Conceptual = semantic/rule violations about clock features or time setting; Stimulus-bound = literal responses (eg, minute hand toward “10” instead of “2” for “ten past”); Perseveration = repetitions/extra elements (eg, >12 numbers or multiple hands); Planning = anchor misplacement (12–3–6–9); Spatial = mis-spacing/mis-ordering not attributable to anchors or neglect; Hemineglect = left hemispace neglect; Numbers outside = numerals written outside the circle; Counterclockwise = reverse numerical sequence. Between-diagnosis differences are interpreted descriptively as distributional differences in error occurrence across diagnoses (see Statistics for handling of multiple-response tables and effect-size reporting). Abbreviations: AD, Alzheimer’s disease; bvFTD, behavioral variant frontotemporal dementia; MCI, mild cognitive impairment; VaD, vascular dementia.

### A simplified six-class scheme

**Figure 4.**
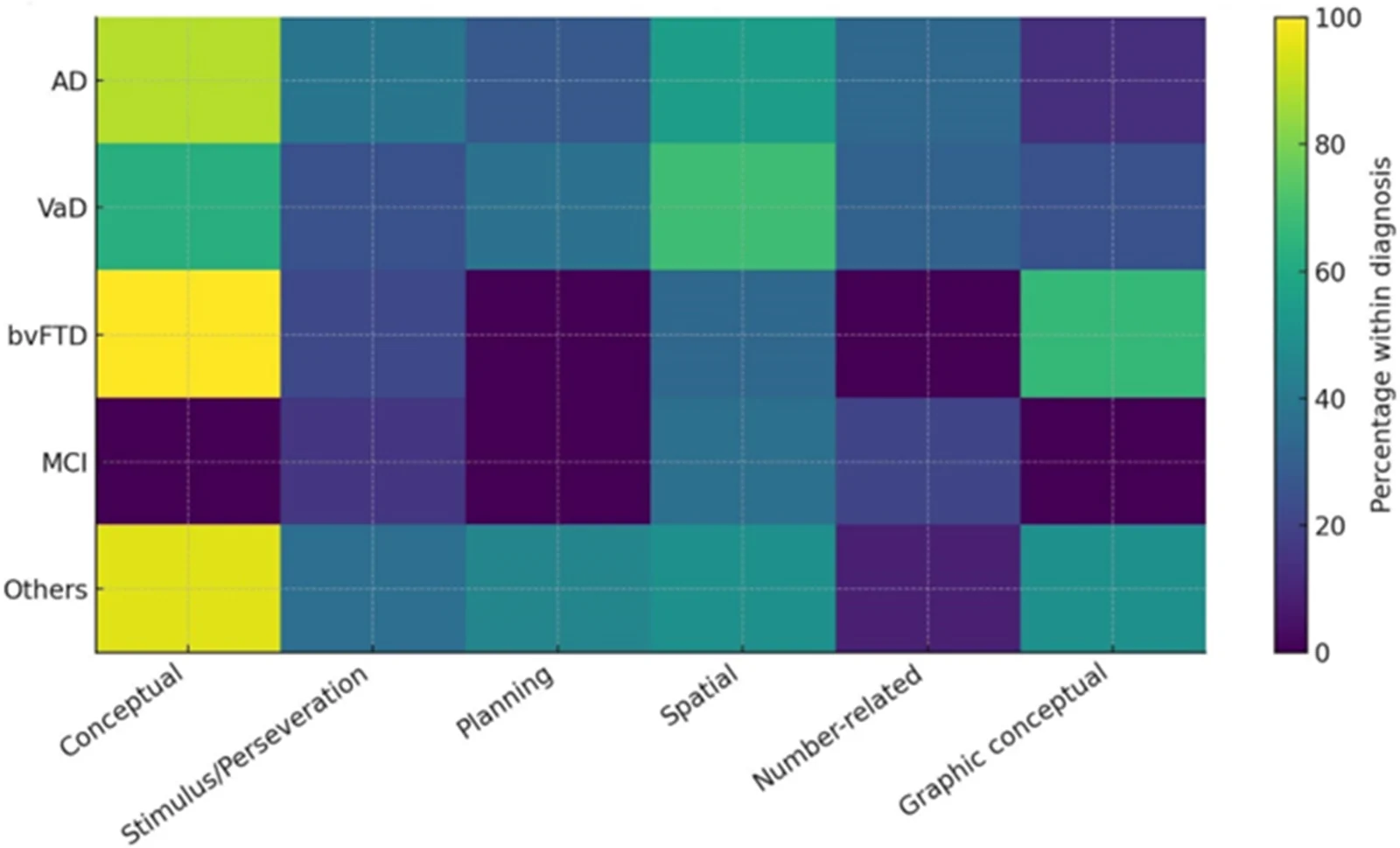
Six-class qualitative error profile by diagnosis. Notes: Heatmap displaying the percentage of patients within each diagnosis meeting each of six qualitative classes: Conceptual, Stimulus-bound/Perseveration, Planning, Spatial, Number-related (outside/counterclockwise), and Graphic conceptual (unrecognizable clock). Colors encode percentage; columns are the six classes; rows (top-to-bottom) are AD, VaD, bvFTD, MCI, Others. Categories are non-mutually exclusive. The six-class framework retained discriminatory power across diagnoses (*χ* ^2^ = 43.365, df = 20, *p* = 0.002). Figure 4. long description.

Collapsing overlapping errors into six classes—conceptual, stimulus-bound/perseveration, spatial, planning, number-related (outside-the-clock/counterclockwise), and graphic-conceptual (unrecognizable clock)—retained a significant distributional association with diagnosis (*χ* ^2^ = 43.365, df = 20, *p* = 0.002; Cramér’s *V* = 0.3). Participants with AD showed mainly conceptual and spatial errors; those with bvFTD combined conceptual errors with frequent graphic-conceptual failures; those with MCI emphasized spatial and number-related errors; and participants with VaD remained heterogeneous (Table 5 and Figure 4). Because these classes are non-exclusive, the chi-square should be interpreted as a distributional summary rather than as a test of mutually exclusive diagnostic categories.

**Table 5.** Simplified Six-Class Scheme by Diagnosis (Row-Percentages; Nonexclusive) Table 5. long description.

Diagnosis (*n*)	Conceptual	Stimulus-bound/Perseveration	Planning	Spatial	Number-related	Graphic conceptual
AD (36)	32 (88.9%)	14 (38.9%)	10 (27.8%)	20 (55.6%)	12 (33.3%)	5 (13.9%)
VaD (16)	10 (62.5%)	4 (25.0%)	6 (37.5%)	11 (68.8%)	5 (31.3%)	4 (25.0%)
bvFTD (9)	9 (100.0%)	2 (22.2%)	0 (0.0%)	3 (33.3%)	0 (0.0%)	6 (66.7%)
MCI (19)	0 (0.0%)	3 (15.8%)	0 (0.0%)	7 (36.8%)	4 (21.1%)	0 (0.0%)
Others (22)	21 (95.5%)	8 (36.4%)	10 (45.5%)	11 (50.0%)	2 (9.1%)	11 (50.0%)

Values are *n* (row %). Classes are not mutually exclusive; within-diagnosis percentages can exceed 100%. Definitions: Conceptual = semantic/rule violations about clock features or time setting; Stimulus-bound/Perseveration = literal time-setting responses (eg, minute hand toward “10” for “ten past”) and/or repeated/extra elements; Planning = anchor misplacement (12–3–6–9); Spatial = spacing/ordering errors not attributable to anchors or neglect; Number-related = outside the circle and/or counterclockwise numbering; Graphic conceptual = unrecognizable clock. When a Graphic conceptual depiction is present, other classes may be indeterminable in that drawing; this class was coded to avoid underestimating the error burden. Pearson’s *χ* ^2^ summarizes distributional differences in error occurrence across diagnoses (*χ^2^* =43.365, df = 20, *p* = 0.002; Cramér’s *V* = 0.3). Abbreviations: AD, Alzheimer’s diseasentia; bvFTD, behavioral variant frontotemporal dementia; MCI, mild cognitive impairment; VaD, vascular deme.

## Discussion

In this tertiary-care cohort from eastern India, qualitative CDT error profiles varied systematically by etiology and provided clinically meaningful information beyond a simple normal/abnormal judgment. The most frequent abnormalities were conceptual and spatial errors, whereas neglect and counterclockwise numbering were uncommon. The proposed six-class framework preserved the main diagnostic signal of established qualitative approaches while offering a more practical structure for drawings with multiple errors or for those in which the clock is too degraded for fine-grained scoring.

Conceptual and graphic-conceptual failures likely reflect disruption of semantic knowledge about clock structure and time-setting rules, together with executive disorganization severe enough to render the drawing globally unrecognizable. In contrast, spatial and planning errors plausibly reflect impairment of visuospatial organization, sequencing, planning, and response monitoring. This interpretation is consistent with the cognitive demands of the CDT, which requires semantic knowledge, visuospatial construction, numerical organization, motor planning, and executive control.^1^ ^–^^5^ ^,^^14^ It also accords with longitudinal and cross-sectional studies showing that conceptual deficits, stimulus-bound behaviors, and perseveration are common in dementia and may become more prominent as impairment advances.^15^ ^,^^16^

The distribution of errors in the present study was coherent with these mechanisms. Participants with AD combined conceptual and spatial errors, in keeping with the dependence of CDT performance on semantic knowledge, visuospatial organization, and executive control, and with prior qualitative studies showing prominent conceptual and spatial disturbances in AD.^4^ ^,^^5^ ^,^^14^ ^–^^20^ Those with bvFTD showed prominent conceptual failures, severe graphic distortion, and frequent omission of fundamental clock elements, consistent with disruption of executive, behavioral, and organizational control systems in frontotemporal syndromes.^11^ ^,^^21^ Participants with VaD displayed heterogeneous performance, as expected given the variable topography of vascular lesions and the frequent contribution of dysfunction in subcortical-frontal circuitry.^22^ In contrast, participants with MCI were characterized mainly by a display of spatial and number-related errors, with comparatively fewer conceptual failures, a pattern consistent with the known limitations of global CDT scoring in milder disease and with the value of qualitative inspection for detecting subtle organizational and visuospatial errors.^1^ ^–^^3^ ^,^^23^ ^–^^25^

Part of the contrast between MCI and the dementia groups may reflect global cognitive severity rather than etiology alone. This is supported by the MMSE and CDR distributions: the MCI group was less globally impaired, whereas the dementia groups showed lower MMSE scores and higher CDR ratings. Therefore, differences such as the higher frequency of correct hand placement in MCI and the more frequent graphic-conceptual failures in bvFTD should be interpreted in light of severity. However, severity alone is unlikely to explain the full pattern. The persistence of distinct qualitative profiles across AD, VaD, bvFTD, MCI, and other etiologies suggests that the type of CDT error also reflects the dominant cognitive systems affected, not merely the overall level of impairment.

Educational background is another important modifier. CDT performance is generally considered useful across a broad range of schooling levels, though subtle features, particularly numeral spacing, sequencing, and hand placement, may still be influenced by education, literacy, and familiarity with analog clocks.^1^ ^–^^3^ ^,^^24^ ^,^^25^ In our cohort, educational attainment ranged from illiteracy to graduate-level education. Although the clock face itself appeared less affected by education, number- and hand-related errors were more frequent among participants with lower educational attainment. This finding supports cautious interpretation of minor number-placement and hand-setting abnormalities, especially in low-education settings. Normative and review data similarly indicate that age and education can influence CDT performance, reinforcing the need to interpret qualitative errors in the context of the patient’s education and culture.^1^ ^–^^3^ ^,^^24^ ^,^^25^

Neglect and counterclockwise numbering were rare and contributed little to distinguishing AD, VaD, and bvFTD in this cohort. These errors are recognized in qualitative CDT frameworks, but their low frequency in our sample suggests limited diagnostic yield for differentiating common neurodegenerative etiologies in routine clinical practice.^4^ ^,^^5^ ^,^^14^ ^,^^26^ Their presence may still be clinically meaningful when interpreted in the broader neurological context, particularly if focal visuospatial or vascular mechanisms are suspected, but they should not be over-weighted as stand-alone markers of dementia subtype.

These observations motivated the development of the simplified six-class framework. Because multiple CDT errors often co-occur, and because some severely impaired drawings cannot be reliably decomposed into all classical categories, the six-class scheme groups related phenomena into clinically interpretable domains: conceptual, stimulus-bound/perseveration, planning, spatial, number-related, and graphic conceptual. Merging stimulus-bound responses with perseveration reflects shared executive-control dysfunction, whereas the graphic-conceptual category captures globally unrecognizable clocks that would otherwise be difficult to score meaningfully. Combining uncommon number-placement anomalies into a single number-related class also improves clinical usability without discarding relevant information. Prior qualitative and comparative studies support the broader principle that CDT errors cluster across cognitive domains and that drawings become increasingly difficult to classify into isolated error types as dementia advances.^4^ ^,^^5^ ^,^^14^ ^–^^16^ ^,^^23^

Several error domains in our study were coded as nonexclusive multiple-response categories. Accordingly, the omnibus *χ* ^2^ tests were used as descriptive summaries of error-occurrence distributions across diagnoses rather than as tests of mutually exclusive patient categories. The observed effect sizes were moderate for clock face and the six-class framework, and large for number- and hand-related errors, supporting the clinical relevance of these qualitative patterns. Nevertheless, the results should be interpreted as pattern recognition within a clinical cohort, not as definitive diagnostic rules.

Clinically, CDT is best used as part of a brief cognitive screening battery rather than as a standalone diagnostic instrument, especially in early disease. Pairing clock drawing with a memory probe, as in the Mini-Cog, improves cognitive screening performance, and recent meta-analytic evidence supports the Mini-Cog’s diagnostic accuracy across healthcare settings.^27^ ^,^^28^ In practice, rapid qualitative appraisal of three elements—overall recognizability of the clock, adequacy of the 12–3–6–9 anchors and number sequence, and presence and correctness of two hands—maps directly onto our six-class framework and may efficiently triage patients for fuller neuropsychological and etiologic assessment.^17^

The proposed framework may be particularly useful in resource-limited settings. In such contexts, a structured qualitative reading of the CDT offers a practical intermediate approach between simple pass/fail scoring and advanced biomarkers or digital tools that may be unavailable.^1^ ^–^^3^ ^,^^27^ ^,^^28^ It is inexpensive, rapid, clinically interpretable, and feasible in busy neurology or primary care settings. Importantly, it should not be presented as a substitute for a full diagnostic evaluation. Rather, it may help clinicians recognize patterns suggestive of different cognitive systems, estimate the need for more detailed assessment, and prioritize patients for neuroimaging, specialist referral, or longitudinal follow-up. This framing is consistent with the Mini-Cog literature, which emphasizes brief, practical screening in time-sensitive and resource-limited settings.^27^ ^,^^28^

Modern digital CDT adds complementary value by quantifying process-level features, including pauses, stroke order, latency, and drawing dynamics, which are invisible in static drawings.^29^ ^–^^32^ Digital CDT studies have shown that such process measures may improve discrimination of MCI or early AD and may correlate with biological measures, including amyloid/tau PET and MRI-derived brain volumes.^29^ ^,^^31^ ^,^^32^ However, digital systems are not universally available. In many real-world settings, especially where specialist neuropsychological testing or biomarker assessment is limited, a structured qualitative approach remains practical and informative.

This study has several strengths, including consecutive recruitment in a tertiary-care Indian setting, a context underrepresented in the CDT literature; qualitative coding anchored to established frameworks; and multilevel analysis across clock components, classical error categories, and a simplified six-class scheme. Limitations should also be acknowledged. The cross-sectional, clinic-based design precludes inference about progression or prediction. The absence of an age- and education-matched cognitively unimpaired control group limits normative interpretation, particularly given the influence of education, literacy, and cultural numeracy on CDT performance.^24^ ^,^^25^ Group sizes were asymmetric, especially for bvFTD, so some within-group percentages have wide uncertainty and should be interpreted cautiously. The “other” etiologies group was clinically heterogeneous. In addition, we did not formally assess inter-rater reliability or diagnostic blinding in the present study. Future studies should include blinded multi-rater coding, reproducibility analyses, larger etiologically balanced samples, and longitudinal follow-up to determine whether specific qualitative CDT patterns predict diagnostic evolution or conversion from MCI to dementia.

In conclusion, qualitative CDT profiles differ meaningfully across cognitive disorders. Conceptual and spatial errors dominate, neglect and counterclockwise numbering are uncommon, and bvFTD is notable for severe graphic-conceptual failures with omission of fundamental clock elements. A condensed six-class qualitative framework preserves clinically relevant information while improving usability when drawings are too degraded for detailed scoring. Used alongside brief memory measures and interpreted in light of severity and education, this approach may support practical, low-cost cognitive assessment in real-world clinical workflows, while complementing digital CDT metrics where such technologies are available.

## Long descriptions

### Table 1. Long description

The table consists of five columns: Diagnosis (n), Normal, Mild distortion, Gross distortion, and Other. Data is presented as n (row percentage).

* A D (36): 20 (55.6 percent) Normal, 12 (33.3 percent) Mild distortion, 3 (8.3 percent) Gross distortion, 1 (2.8 percent) Other.

* V a D (16): 8 (50.0 percent) Normal, 8 (50.0 percent) Mild distortion, 0 (0.0 percent) Gross distortion, 0 (0.0 percent) Other.

* b v F T D (9): 0 (0.0 percent) Normal, 5 (55.6 percent) Mild distortion, 4 (44.4 percent) Gross distortion, 0 (0.0 percent) Other.

* M C I (19): 18 (94.7 percent) Normal, 0 (0.0 percent) Mild distortion, 0 (0.0 percent) Gross distortion, 1 (5.3 percent) Other.

* Others (22): 5 (22.7 percent) Normal, 15 (68.2 percent) Mild distortion, 2 (9.1 percent) Gross distortion, 0 (0.0 percent) Other.

Statistical notes indicate a Pearson chi-squared value of 47.306, d f equals 12, p less than 0.001, and Cramer’s V equals 0.4. Other denotes non-circular or incomplete outlines.

Navigate back to Table 1..

### Figure 1. Long description

The y-axis represents the Percentage of patients from 0 to 100. The x-axis lists five diagnostic groups. A legend at the bottom left identifies four categories: Normal (orange), Mild distortion (light blue), Gross distortion (green), and Other (yellow).

* A D: Approximately 55 percent Normal, 34 percent Mild distortion, 8 percent Gross distortion, and 3 percent Other.

* V a D: 50 percent Normal and 50 percent Mild distortion.

* b v F T D: Approximately 55 percent Mild distortion and 45 percent Gross distortion. This group has 0 percent Normal outcomes.

* M C I: Approximately 95 percent Normal and 5 percent Other.

* Others: Approximately 23 percent Normal, 68 percent Mild distortion, and 9 percent Gross distortion.

The M C I group shows the highest proportion of Normal outcomes, while b v F T D shows the highest proportion of Gross distortion and no Normal outcomes.

Navigate back to Figure 1..

### Table 2. Long description

The table consists of 9 columns and 6 rows of data. The columns are: Diagnosis (n), No numbers, Missing numbers, Added (>12), Spatial errors, Counterclockwise, Outside the circle, Other/multiple language, and Perfect numbering.

* A D (36): 11 (30.6%) No numbers, 16 (44.4%) Missing numbers, 3 (8.3%) Added, 11 (30.6%) Spatial errors, 2 (5.6%) Counterclockwise, 10 (27.8%) Outside the circle, 4 (11.1%) Other/multiple language, 2 (5.6%) Perfect numbering.

* VaD (16): 5 (31.3%) No numbers, 5 (31.3%) Missing numbers, 1 (6.3%) Added, 9 (56.3%) Spatial errors, 1 (6.3%) Counterclockwise, 4 (25.0%) Outside the circle, 2 (12.5%) Other/multiple language, 0 (0.0%) Perfect numbering.

* bvFTD (9): 7 (77.8%) No numbers, 2 (22.2%) Missing numbers, 0 (0.0%) Added, 2 (22.2%) Spatial errors, 0 (0.0%) Counterclockwise, 0 (0.0%) Outside the circle, 0 (0.0%) Other/multiple language, 0 (0.0%) Perfect numbering.

* M C I (19): 0 (0.0%) No numbers, 0 (0.0%) Missing numbers, 0 (0.0%) Added, 7 (36.8%) Spatial errors, 1 (5.3%) Counterclockwise, 3 (15.8%) Outside the circle, 0 (0.0%) Other/multiple language, 10 (52.6%) Perfect numbering.

* Others (22): 9 (40.9%) No numbers, 11 (50.0%) Missing numbers, 2 (9.1%) Added, 6 (27.3%) Spatial errors, 0 (0.0%) Counterclockwise, 2 (9.1%) Outside the circle, 1 (4.5%) Other/multiple language, 0 (0.0%) Perfect numbering.

* Total (102): 32 No numbers, 34 Missing numbers, 6 Added, 35 Spatial errors, 4 Counterclockwise, 19 Outside the circle, 7 Other/multiple language, 12 Perfect numbering.

Navigate back to Table 2..

### Figure 2. Long description

The y-axis is labeled Percentage of patients and ranges from 0 to 80. The x-axis lists five diagnostic groups: A D, V a D, b v F T D, M C I, and Others. A legend in the top right identifies four categories: Perfect numbering (orange), Spatial errors (light blue), Missing numbers (green), and No numbers (yellow).

* A D: Perfect numbering is lowest at approximately 5 percent. Missing numbers is highest at approximately 45 percent. Spatial errors and No numbers are both around 30 percent.

* V a D: Spatial errors are highest at approximately 56 percent. Missing numbers and No numbers are both around 31 percent. Perfect numbering is 0 percent.

* b v F T D: No numbers is the dominant outcome at approximately 78 percent. Spatial errors and Missing numbers are both around 22 percent. Perfect numbering is 0 percent.

* M C I: Perfect numbering is highest at approximately 53 percent. Spatial errors are around 37 percent. Missing numbers and No numbers are 0 percent.

* Others: Missing numbers is highest at approximately 50 percent, followed by No numbers at 41 percent and Spatial errors at 27 percent. Perfect numbering is 0 percent.

Navigate back to Figure 2..

### Table 3. Long description

The table consists of six columns: Diagnosis (n), No hands, Single hand, Two hands (improper), Perseveration, and Correct. Data is presented as count followed by row percentage.

* A D (36): 13 (36.1 percent) No hands, 5 (13.9 percent) Single hand, 8 (22.2 percent) Two hands (improper), 9 (25.0 percent) Perseveration, 0 (0.0 percent) Correct.

* V a D (16): 5 (31.3 percent) No hands, 2 (12.5 percent) Single hand, 5 (31.3 percent) Two hands (improper), 2 (12.5 percent) Perseveration, 0 (0.0 percent) Correct.

* b v F T D (9): 8 (88.9 percent) No hands, 0 (0.0 percent) Single hand, 0 (0.0 percent) Two hands (improper), 1 (11.1 percent) Perseveration, 0 (0.0 percent) Correct.

* M C I (19): 0 (0.0 percent) No hands, 0 (0.0 percent) Single hand, 4 (21.1 percent) Two hands (improper), 2 (10.5 percent) Perseveration, 13 (68.4 percent) Correct.

* Others (22): 14 (63.6 percent) No hands, 0 (0.0 percent) Single hand, 5 (22.7 percent) Two hands (improper), 4 (18.2 percent) Perseveration, 0 (0.0 percent) Correct.

Footnote: Correct hand placement occurred only in the M C I group. Pearson chi-squared equals 83.618, d f equals 16, p is less than 0.001.

Navigate back to Table 3..

### Figure 3. Long description

A grouped bar chart with the Y-axis labeled Percentage of patients from 0 to 80 and the X-axis listing five diagnostic groups. A legend in the top right identifies five categories: No hands (orange), Single hand (light blue), Two hands improper (green), Perseveration (yellow), and Correct (dark blue).

* A D: No hands is approximately 36 percent, Single hand is 14 percent, Two hands improper is 22 percent, and Perseveration is 25 percent.

* V a D: No hands is 31 percent, Single hand is 12 percent, Two hands improper is 31 percent, and Perseveration is 12 percent.

* b v F T D: No hands is the highest at nearly 90 percent, with Perseveration at approximately 11 percent. Other categories are absent.

* M C I: Two hands improper is 21 percent, Perseveration is 10 percent, and Correct is the highest at 68 percent. No hands and Single hand are absent.

* Others: No hands is 64 percent, Two hands improper is 23 percent, and Perseveration is 18 percent. Single hand and Correct are absent.

Navigate back to Figure 3..

### Table 4. Long description

The table consists of 9 columns and 5 diagnostic rows. The columns are: Diagnosis n, Conceptual, Perseveration, Stimulus-bound, Planning, Spatial, Hemineglect, Numbers outside, and Counterclockwise.

Row 1: A D n equals 36. Conceptual 32 88.9 percent, Perseveration 10 27.8 percent, Stimulus-bound 4 11.1 percent, Planning 10 27.8 percent, Spatial 20 55.6 percent, Hemineglect 0 0.0 percent, Numbers outside 10 27.8 percent, Counterclockwise 2 5.6 percent.

Row 2: V a D n equals 16. Conceptual 10 62.5 percent, Perseveration 3 18.8 percent, Stimulus-bound 1 6.3 percent, Planning 6 37.5 percent, Spatial 9 56.3 percent, Hemineglect 2 12.5 percent, Numbers outside 4 25.0 percent, Counterclockwise 1 6.3 percent.

Row 3: b v F T D n equals 9. Conceptual 9 100.0 percent, Perseveration 2 22.2 percent, Stimulus-bound 0 0.0 percent, Planning 0 0.0 percent, Spatial 2 22.2 percent, Hemineglect 1 11.1 percent, Numbers outside 0 0.0 percent, Counterclockwise 0 0.0 percent.

Row 4: M C I n equals 19. Conceptual 0 0.0 percent, Perseveration 2 10.5 percent, Stimulus-bound 1 5.3 percent, Planning 0 0.0 percent, Spatial 7 36.8 percent, Hemineglect 0 0.0 percent, Numbers outside 3 15.8 percent, Counterclockwise 1 5.3 percent.

Row 5: Others n equals 22. Conceptual 21 95.5 percent, Perseveration 8 36.4 percent, Stimulus-bound 0 0.0 percent, Planning 10 45.5 percent, Spatial 10 45.5 percent, Hemineglect 1 4.5 percent, Numbers outside 2 9.1 percent, Counterclockwise 0 0.0 percent.

Navigate back to Table 4..

### Table 5. Long description

The table consists of seven columns. The first column lists the Diagnosis and sample size n. The subsequent six columns represent error classes: Conceptual, Stimulus-bound or Perseveration, Planning, Spatial, Number-related, and Graphic conceptual. Data is presented as n followed by row percentage.

* A D (36): Conceptual 32 (88.9%), Stimulus-bound 14 (38.9%), Planning 10 (27.8%), Spatial 20 (55.6%), Number-related 12 (33.3%), Graphic conceptual 5 (13.9%).

* V a D (16): Conceptual 10 (62.5%), Stimulus-bound 4 (25.0%), Planning 6 (37.5%), Spatial 11 (68.8%), Number-related 5 (31.3%), Graphic conceptual 4 (25.0%).

* b v F T D (9): Conceptual 9 (100.0%), Stimulus-bound 2 (22.2%), Planning 0 (0.0%), Spatial 3 (33.3%), Number-related 0 (0.0%), Graphic conceptual 6 (66.7%).

* M C I (19): Conceptual 0 (0.0%), Stimulus-bound 3 (15.8%), Planning 0 (0.0%), Spatial 7 (36.8%), Number-related 4 (21.1%), Graphic conceptual 0 (0.0%).

* Others (22): Conceptual 21 (95.5%), Stimulus-bound 8 (36.4%), Planning 10 (45.5%), Spatial 11 (50.0%), Number-related 2 (9.1%), Graphic conceptual 11 (50.0%).

Navigate back to Table 5..

### Figure 4. Long description

A heatmap grid with five rows representing diagnoses and six columns representing error classes. The y-axis lists diagnoses from top to bottom as A D, V a D, b v F T D, M C I, and Others. The x-axis lists error classes from left to right as Conceptual, Stimulus/Perseveration, Planning, Spatial, Number-related, and Graphic conceptual.

A color scale on the right indicates percentages from 0 (dark purple) to 100 (bright yellow).

Key data trends include:

* Conceptual errors show the highest prevalence across most groups, appearing bright yellow for b v F T D and Others, and light green for A D and V a D, while M C I shows a dark purple low prevalence.

* Spatial errors are moderately prevalent across all groups, indicated by teal and green shades.

* Planning and Number-related errors show the lowest prevalence in the b v F T D and M C I groups, indicated by dark purple cells.

* Graphic conceptual errors are most prominent in the b v F T D group compared to other diagnoses.

Navigate back to Figure 4..
